# Evaluation of carotid intima media thickness in children with idiopathic nephrotic syndrome

**DOI:** 10.1186/s13052-022-01383-7

**Published:** 2022-12-09

**Authors:** Ashraf Sayed Kamel, Mohamed Mohamed Ezzat AlGhawass, Muhammad Adel Sayed, Sara Aly Roby

**Affiliations:** 1 Pediatrics department, Faculty of Medicine, Fayoum University, Fayoum city, Egypt; 2 Radiology department, Faculty of Medicine, Fayoum University, Fayoum city, Egypt

**Keywords:** Nephrotic syndrome, Children, Carotid intima media thickness, Dyslipidemia

## Abstract

**Background:**

Nephrotic syndrome is the one of the commonest renal disorders in children. Children with nephrotic syndrome (NS) are at a high risk of atherosclerosis due to hyperlipidemia, hypertension. Carotid intima media thickness (CIMT) is a surrogate marker for atherosclerosis. This study aimed to evaluate the carotid intima media thickness in children with nephrotic syndrome and its relation to different risk factors.

**Methods:**

This is an observational case control study that included forty children with nephrotic syndrome and thirty healthy children as controls. The inclusion criteria were: age of 2 years or more with disease duration of minimum of 1 year and glomerular filtration rate > 90 mL/min/1.73m^2^. CIMT was assessed by ultrasound. Lipid profile, protein/creatinine ratio in urine and kidney function tests were done.

**Results:**

The mean CIMT (mm) was significantly higher in patients with NS (0.477 ± 0.04) compared to controls (0.39 ± 0.03) (*P* < 0.001) even when compared across different age groups. 60% of patients had received non-steroid immunosuppressive therapy. CIMT was significantly higher in patients receiving non-steroid immunosuppressive therapy than those receiving steroids alone. Subsequently, CIMT had significant positive correlation to duration of the disease (*p* = 0.05), body mass index (BMI) (*p* = 0.03), number of relapses (*p* = 0.01) and diastolic blood pressures (*p* = 0.003).

**Conclusion:**

Children with NS had significantly higher CIMT than control group. CIMT was positively correlated to disease duration, number of relapses and BMI. It was significantly higher among patients receiving non-steroid immunosuppressive therapy than those receiving steroids alone.

## Background

Carotid intima media thickness (CIMT) is a reliable marker of atherosclerosis and its complications in adults, such as myocardial infarction and stroke [1, 2]. Its implication in children is still debatable however there are increasing numbers of studies in children with risk factors for vascular injury [3].

Nephrotic syndrome (NS) is one of the commonest renal disorders in children [4]. The clinical characteristics of NS include massive proteinuria, hypoalbuminemia and generalized edema [5]. It is associated with an atherogenic lipoprotein profile with increased total serum cholesterol and low-density lipoprotein (LDL) [6, 7].

Disturbances of lipid metabolism in NS might be persistent even during remission periods [8–10] and may predispose to endothelial dysfunction and structural atherosclerosis [10]. It is also well established that atherosclerosis often begins during childhood [11].

There are only few studies that evaluated CIMT in children with idiopathic nephrotic syndrome. This study was designed to evaluate the CIMT in children with idiopathic nephrotic syndrome as an atherosclerosis surrogate marker in children with nephrotic syndrome and assess different risk factors that could affect CIMT.

## Methods

This observational case control study was carried out in the period from November 2021 till July 2022. Forty children with idiopathic NS following up at the pediatric-nephrology outpatient clinic of Fayoum university hospital were included in the study. Patients were categorized according to their response to steroid treatment into 3 groups; steroid-sensitive (SSNS), steroid-dependent (SDNS) and steroid-resistant (SRNS). Thirty children with history of minor non-renal illness who attended the general outpatient clinic were included as a control group. Children of both groups were age and sex-matched.

The Inclusion criteria were: Age is 2 to 18 years old at time of enrollment in the study, being on continuous or interrupted treatment for at least 1 year before enrollment and glomerular filtration rate more than 90 mL/min/1.73m^2^. The exclusion criteria were: patients having history of familial hypercholesterolemia, known case of essential hypertension, children with type-1 diabetes mellitus or obesity, presence of edema at time of examination.

All children were subjected to full history taking and complete physical examination stressing on age at diagnosis, duration of the disease, response to steroid therapy, number of relapses, immunosuppressive drugs, anthropometric measurements including weight, height, body mass index (BMI) and arterial blood pressure measurement. Laboratory investigations were done including serum albumin, urea and creatinine level, spot albumin/creatinine ratio in urine (ACR), total serum cholesterol, and triglycerides (TG) levels. Hypercholesterolemia was defined as serum cholesterol level at or above 200 mg/dL, hypertriglyceridemia as serum triglyceride level at or above 100 mg/dL (children aged < 9 years) or above 130 mg/dL (children aged 10–18 years). glomerular filtration rate (GFR) was calculated using Schwartz formula [12].

Patients were classified according to the following definitions of nephrotic syndrome:

Remission: 24 hours’ urinary proteins less than 4 mg/m2/h, nil or trace by dipstick for at least three consecutive days.

Relapse: 24 hours urinary proteins more than 40 mg/m2/h or urinary proteins more than 3+ by dipstick in for at least three consecutive days.

Infrequent relapse nephrotic syndrome (IFRNS): 3 or less relapses within one year,

Frequent relapse nephrotic syndrome (FRNS): Two or more relapses within 6 months of steroid-induced remission; 4 or more relapses in any 12 months period.

Steroid dependent nephrotic syndrome (SDNS): Occurrence of two consecutive relapses during alternate day steroid therapy or within 2 weeks of its discontinuation;

Steroid resistant nephrotic syndrome (SRNS): Failure to achieve remission after 4–6 weeks of daily oral prednisolone therapy at a dose of 2 mg/kg/day.

Both infrequently and frequently relapsing patients were grouped into “steroid sensitive nephrotic syndrome (SSNS)” in this study.

### CIMT measurement

Measurement of CIMT was performed according to Mannheim’s Consensus Guideline 2011 [13] by an experienced radiologist who was blinded to the patient’s condition. The ultrasound measurement is obtained using a single ultrasound machine (General Electric, LOGIQ *e*) with a linear high-frequency transducer (7.5 MHz). The CIMT is defined as the distance between the first echogenic line (lumen–intima interface) and the second echogenic line (media–adventitia interface) of the far wall, using a manual cursor placement measurement technique. This double-line pattern is seen by 2D- echo on both walls of the common carotid artery (CCA) in the longitudinal view.

**Fig. 1:**
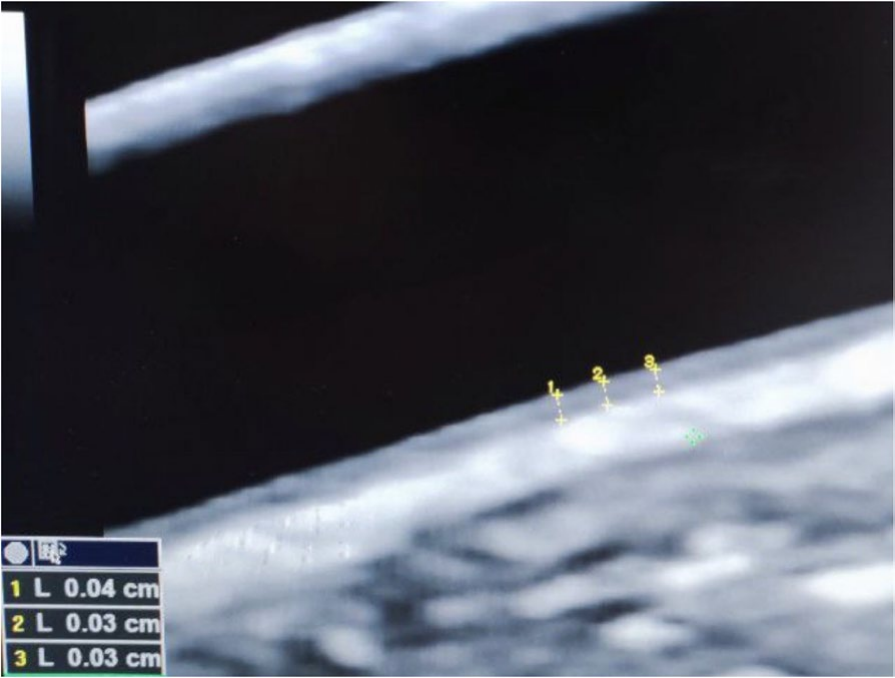
measurement of CIMT in one of controls

The examination was performed after 10 minutes of rest with the patient at supine position with a slightly overextended neck and head turned slightly to the contralateral side. Measurements are obtained from the far wall of the common carotid artery on both sides at 10–20 mm proximal to the bifurcation. On the Images of the thickest CIMT, measurements were taken with calipers positioned on a zoomed image of the common carotid artery. CIMT was measured at end diastole. The Mean value of the 3 measurements on each side was used (Fig. 1).

Data analysis was performed using the Statistical Package of Social Science (SPSS) software version 22 in windows 7 (SPSS Inc., Chicago, IL, USA). For qualitative data, Chi-square test was used to compare between two or more qualitative groups. For quantitative data, Independent samples *t*-test and one-way ANOVA test, Krystal Wallis test and Mann-Whitney test were used. Correlations between the study’s continuous variables were done using the Pearson correlation test. The *P*-value ≤0.05 was considered as statistically significant.

## Results

This study included 40 children with idiopathic nephrotic syndrome and 30 age-and sex-matched healthy children as controls. Children were stratified according to their age at time of the study into 3 groups: G1; ≤5 years (9 patients and 10 controls), G2; 6–10 years (17 patients, 13 controls), and G3; > 10 years (14 patients and 7 controls). The clinical and laboratory data of the study groups are shown in Table 1.

**Table 1:** Clinical and laboratory data of cases and controls

Variables		Cases (*N* = 40)		Control (*N* = 30)		*P*-value	Sig.
		Mean	SD	Mean	SD		
Age at time of study (years)		8.5	3.5	7.03	3.3	0.07	NS
Sex	Male	29	72.5%	18	60%	0.3	NS
	Female	11	27.5%	12	40%		
Weight (kg)		28.7	11.6	22.1	8.6	**0.01**	**S**
Height (cm)		124.1	18.9	116.1	20.1	0.09	NS
Age groups	G1: ≤5 years	9	22.5%	10	33.3%	0.4	NS
	G2: 6–10 years	17	42.5%	13	43.3%		
	G3: > 10 years	14	35%	7	23.4%		
BMI (kg/m^2^)		17.9	2.8	15.8	2.2	**0.001**	**HS**
Blood Pressure	Systolic BP (mmHg)	103.6	10.5	97.1	12.1	**0.01**	**S**
	Diastolic BP (mmHg)	67.9	8.4	64.5	6.5	0.07	NS
Hypertension	Normal	22	55%	30	100%	**< 0.001**	**HS**
	Pre-hypertension	4	10%	0	0%		
	Stage 1 hypertension	12	30%	0	0%		
	Stage 2 hypertension	2	5%	0	0%		
RBS (mg/dL)		93.6	13.1	92.4	12.3	0.7	NS
Serum albumin (gm/dL)		4.1	0.73	4.4	0.35	0.1	NS
Serum Urea (mg/dL)		30.7	11.9	25.7	8.3	0.06	NS
Serum Creatinine (mg/dL)		0.53	0.16	0.51	0.14	0.6	NS
GFR		139.9	46.5	131.6	34.4	0.4	NS
Total Cholesterol (mg/dL)		216.2	95.2	142.1	16.8	**< 0.001**	**HS**
TG (mg/dL)		134.4	102.9	93.2	19.8	**0.03**	**S**

Abbreviations: *BMI* body mass index, *BP* blood pressure, *GFR* glomerular filtration rate, *RBS* Random blood sugar, *TG* triglycerides

In the patients’ group, 10 (25%) patients were steroid-sensitive nephrotic syndrome (SSNS) which included frequently and infrequently relapsing patients, 20 (50%) were steroid-dependent nephrotic syndrome (SDNS) and 10 (25%) were steroid-resistant nephrotic syndrome (SRNS). The mean age at disease onset was 5.4 ± 2.9 years (range 1.3–16 years). The mean disease duration was 3.2 ± 2.5 years (range 1–10 years) and the mean number of relapses was 3 ± 2 (range 0–8 relapses) among cases.

There was no statistically significant difference between cases and controls as regards the age and sex. The weight and BMI were significantly higher in nephrotic patients as compared to the healthy controls (*p*-value = 0.01 and 0.001 respectively). On the other hand, there was no statistically significant difference as regards the height.

There was a significantly higher mean systolic blood pressure in the cases group (*p*-value = 0.01) as compared to controls. However, there was no statistically significant difference as regards the mean diastolic blood pressure. Also significantly higher percentage of stage 1 and 2 hypertension was found among cases as compared to controls (*p* value< 0.001). Among the nephrotic patients, 35% were found to have hypertension (stage 1; 30% and stage 2; 5%).

The mean total serum cholesterol and triglyceride levels were significantly higher among cases than controls with *p*-value < 0.001 and 0.03 respectively. On the other hand, there was no statistically significant difference as regards other laboratory investigations (random blood sugar, serum albumin, serum urea, creatinine or GFR). In this study, 17 out of 40 patients (42.5%) had hypercholesterolemia but none of controls had (*p* < 0.001). Similarly, 17 patients (42.5%) had hypertriglyceridemia compared to 3 children (10%) in the control group (*p* = 0.003).

Among our patients, 60% had received non steroid-immunosuppressive agent. 30% of patients had received calcineurin inhibitors (CNI), 15% received mycophenolate mofetil (MMF), 2.5% received cyclophosphamide (CYP), 12.5% received combined therapy (MMF, CNI and CYP).

Among patients with SRNS and SDNS, 11 patients had underwent renal biopsy; 4 patients had minimal change disease (MCD), 4 patients had focal segmental glomerulosclerosis (FSGS), 2 patients had mesangiocapillary glomerulonephritis and 1 patient had membranous glomerulonephritis (GN).

The clinical and laboratory data of the three nephrotic groups are shown in Table 2. Patients with SDNS had significantly higher mean duration of the disease (*p*-value = 0.01), number of relapses (*p*-value< 0.001) and mean CIMT (*p*-value = 0.03) than the other groups. However, patients with SRNS had significantly higher mean ACR (*p*-value = 0.009). On the other hand, there was no statistically significant difference between the 3 groups of nephrotic syndrome (*p*-value> 0.05) as regards other clinical and laboratory data.

**Table 2:** Clinical and laboratory Data of the Three Nephrotic Groups

Variables	SSNS (*n* = 10)	SDNS (*n* = 20)	SRNS (*n* = 10)	*P*-value	Sig.
	Mean ± SD	Mean ± SD	Mean ± SD		
Age (years)	7.3 ± 2.6	9.6 ± 3.7	7.6 ± 3.4	0.1	NS
Weight (kg)	25.8 ± 6.9	32.7 ± 13.4	23.9 ± 9.2	0.09	NS
Height (cm)	121.4 ± 16.4	129.6 ± 19.7	115.5 ± 17.8	0.1	NS
BMI (kg/m2)	17.3 ± 2.1	18.6 ± 2.9	17.3 ± 3.3	0.4	NS
Age at disease onset (years)	4.4 ± 3.3	5.6 ± 2.5	6 ± 3.2	0.5	NS
Disease duration (years)	2.8 ± 0.95	4.2 ± 3.1	1.6 ± 0.95	**0.01**	**S**
Number of relapses	3 ± 1	4 ± 2	1 ± 1	**< 0.001**	**HS**
Systolic pressure (mmHg)	99.5 ± 7.5	104.6 ± 12.1	105.7 ± 9.03	0.4	NS
Diastolic pressure (mmHg)	62.7 ± 3.2	69.8 ± 9.2	69.5 ± 8.6	0.07	NS
RBS (mg/dL)	89.9 ± 11.7	97.7 ± 12.9	89 ± 13.2	0.1	NS
Serium albumin (mg/dL)	4.6 ± 0.55	4.02 ± 0.76	3.9 ± 0.70	0.08	NS
Urea (mg/dL)	31.3 ± 5.8	31.2 ± 15.1	29 ± 9.8	0.9	NS
Creatinine (mg/dL)	0.57 ± 0.14	0.54 ± 0.19	0.46 ± 0.11	0.2	NS
GFR	121.7 ± 24.7	146.7 ± 54.1	144.9 ± 45.9	0.4	NS
Total Cholesterol (mg/dL)	180 ± 55.8	223 ± 102.7	238.7 ± 108.3	0.4	NS
TG (mg/dL)	115.7 ± 90.2	143.7 ± 99.2	134.5 ± 128.1	0.4	NS
ACR (g/g creatinine)	0.099 ± 0.12	0.431 ± 0.75	2.67 ± 4.6	**0.009**	**HS**
CIMT (mm)	0.453 ± 0.04	0.495 ± 0.04	0.466 ± 0.03	**0.03**	**S**

Abbreviations: *ACR* albumin/creatinine ratio in urine, *BMI* body mass index, *BP* blood pressure, *CIMT* carotid intima media thickness, *GFR* glomerular filtration rate, *RBS* Random blood sugar, *SDNS* steroid dependent NS, *SRNS* steroid resistant NS, *SSNS* steroid sensitive nephrotic syndrome, *TG* triglycerides

On comparing the two study groups regarding the CIMT, it was significantly higher in patients (mean of 0.477 mm) as compared to controls (mean of 0.391 mm) with *p* value< 0.001 (Table 3). CIMT remained significantly higher in patients as compared to controls across the all three age groups with *p*- value of 0.001 (Table 4). However, on comparing patients according to renal biopsy results, there was no significant difference regarding the CIMT (Table 5).

**Table 3:** Carotid Intima Media Thickness in cases and controls

Parameter	CIMT (mm)		*P*-value	Sig.
	Mean	SD		
Cases (*n* = 40)	0.477	0.04	**< 0.001**	**HS**
Controls (*n* = 30)	0.391	0.03		

Abbreviations: *CIMT* carotid intima media thickness

**Table 4:** Carotid Intima Media Thickness According to Age Groups in Cases and Controls

Age groups	CIMT (mm) in Cases (*N* = 40)		CIMT (mm) in Control (*N* = 30)		*P*-value	Sig.
	Mean	SD	Mean	SD		
≤5 years	0.462	0.05	0.382	0.03	**0.001**	**HS**
6–10 years	0.467	0.04	0.40	0.03	**0.001**	**HS**
> 10 years	0.499	0.03	0.388	0.05	**0.001**	**HS**

Abbreviations: *CIMT* carotid intima media thickness

**Table 5:** Comparison of CIMT in different renal biopsy results among cases

Renal biopsy	CIMT (mm)		*P*-value	Sig.
	Mean	SD		
MCD	0.483	0.07	0.9	NS
FSGS	0.485	0.01		
Membranous GN	0.48	0		
Mesangiocapillary GN	0.485	0.04		

Abbreviations: *CIMT* carotid intima media thickness, *FSGS* focal segmental glomerulosclerosis, *GN* glomerulonephritis, *MCD* minimal change disease

We found that the CIMT was significantly higher among patients with nephrotic syndrome receiving non-steroid immunosuppressive therapy (*n* = 24) than those not receiving therapy (*n* = 16) (0.494 ± 0.04 versus 0.452 ± 0.04; *P*-value = 0.003) as shown in Table 6. There was also a statistically significant higher mean CIMT among cases who received combined or serially CYP, CNI & MMF (*p*-value = 0.04). However, there was no significant difference in CIMT between patients treated with CNI (*n* = 17) and those that were not treated with CNI (*n* = 23) (0.492 ± 0.05 versus 0.466 ± 0.04; *P*-value = 0.07).

**Table 6:** Comparison of CIMT in different treatment regimens among cases

Immunosuppressant	CIMT (mm)		*P*-value	Sig.
	Mean	SD		
Treatment regimen				
Steroids alone (*n* = 16)	0.452	0.04	**0.003**	**HS**
Non-steroid Immunosuppressive agents (*n* = 24)	0.494	0.04		
Types of treatment				
Steroids alone	0.452	0.04	**0.04**	**S**
CNI	0.483	0.05		
MMF	0.493	0.03		
CYP	0.52	0		
MMF &CNI	0.50	0.04		
CYP &CNI & MMF	0.535	0.05		
Treatment by CNI				
Not on CNI (*n* = 23)	0.466	0.04	0.07	NS
On CNI (*n* = 17)	0.492	0.05		

Abbreviations: *CIMT* carotid intima media thickness, *CNI* calcineurin inhibitors, *CYP* cyclophosphamide, *MMF* mycophenolate mofetil

Correlations between CIMT and other clinical and laboratory data are shown in Table 7. We found a statistically significant positive correlation between CIMT and patients’ age *(r = 0.45, p = 0.004)*, patients’ weight (*r = 0.42, p = 0.007*), height (*r = 0.36, p = 0.02*), BMI *(r = 0.33, p = 0.03)*, age at disease onset (*r = 0.33, p = 0.03*), duration of the disease (*r = 0.31, p = 0.05*), number of relapses (*r = 0.38, p = 0.01*) and diastolic blood pressure (*r = 0.45, p = 0.003*). However, there were no significant correlations between CIMT and systolic blood pressure, total cholesterol, triglycerides, serum urea, serum creatinine or GFR. A negative, but statistically insignificant correlation was noted between CIMT and serum albumin *(r = − 0.22, p > 0.05)* and albumin/creatinine ratio in urine *(r = − 0.09, p > 0.05).*

**Table 7:** Correlation between CIMT and Other Clinical and Laboratory Data among cases

Variables	CIMT (mm)		
	r	*P*-value	Sig.
Age	**0.45**	**0.004**	**HS**
Weight (kg)	**0.42**	**0.007**	**HS**
Height (cm)	**0.36**	**0.02**	**S**
BMI (kg/m2)	**0.33**	**0.03**	**S**
Age at disease onset (years)	**0.33**	**0.03**	**S**
Disease duration (years)	**0.31**	**0.05**	**S**
Number of relapses	**0.38**	**0.01**	**S**
Systolic BP	0.28	0.08	NS
Diastolic BP	**0.45**	**0.003**	**HS**
Serium albumin (mg/dL)	−0.22	0.2	NS
Urea (mg/dL)	0.007	0.9	NS
Creatinine (mg/dL)	−0.05	0.7	NS
GFR	0.27	0.09	NS
Total Cholesterol (mg/dL)	0.05	0.8	NS
TG (mg/dL)	0.06	0.7	NS
ACR (g albumin/g creatinine)	−0.09	0.6	NS

Abbreviations: *ACR* albumin creatinine ratio in urine, *BMI* body mass index, *BP* blood pressure, *CIMT* carotid intima media thickness, *GFR* glomerular filtration rate, *TG* triglycerides

## Discussion

This study was designed to evaluate the CIMT in children with idiopathic nephrotic syndrome and its correlation with dyslipidemia and other risk factors.

In this study, the body weight and BMI were significantly higher in NS patients as compared to the healthy controls (*p*-value = 0.01 and 0.001 respectively). Similar results were reported by other studies [14–18]. This finding can be attributed to the excess weight gain during treatment with steroids which can persist even after its discontinuation and usually associated with dyslipidemia that can increase the cardiovascular risk [19].

In our study, the mean systolic blood pressure was significantly higher in patients than controls but no significant difference in the mean diastolic blood pressure was found between patients and controls. However, Paripović et al. [20] found no differences in both mean systolic and diastolic blood pressure between the two groups. Hooman et al. [21] and Chaubey et al. [22] found significantly higher both mean systolic and diastolic blood pressure in patients than controls.

Among our patients, 35% were found to have hypertension as compared to 12% of patients reported by Ahmed et al. [18].

Total serum cholesterol and triglyceride levels were significantly higher in cases than controls. Similar results were reported by other studies [10, 17]. High lipid profile could have a role in the pathogenesis of increased oxidative stress in children with NS through synthesis of atherogenic factors such as malondialdehyde [23].

In this study, 17 out of 40 patients (42.5%) had high total serum cholesterol (> 200 mg/dL) and TG levels. Paripović et al. [20] reported similar percentage of patients having hypercholesterolemia (17 out of 40 patients; 42.5%) but a slightly lower percentage having high TG level (35%). Similarly, in a study by Candan et al. [24] high total cholesterol (> 200 mg/dL) was noted in 54% of patients.

On stratification of the patients according to their response to steroids, patients with SDNS had significantly higher mean duration of the disease (*p*-value = 0.01), number of relapses (*p*-value< 0.001). However, Ahmed et al. [18] didn’t find any significant difference between their NS groups as regards the mean duration of disease. In our study, patients with SRNS had significantly higher mean albumin creatinine ratio in urine (ACR) (*p*-value = 0.009) which is in agreement with the findings by Ahmed et al. [18].

CIMT is a marker for the evaluation of atherosclerosis secondary to risk factors as hypertension, hyperlipidemia, and endothelial dysfunction [25]. In the current study, CIMT was significantly higher in children with NS as compared to controls across all age groups. Similar results were reported by other studies [17, 18, 21, 24]. Correspondingly, Mehta et al. [26] in their study, conducted on 66 children with NS and 128 age and sex matched healthy controls, found that CIMT was significantly higher in NS especially in the age above 4 years. In consonance with our study, Kari et al. [27] reported higher CIMT in children with SRNS than the controls.

However, unlike our results, Kniazewska et al. [10] didn’t find significant differences in CIMT between 30 children previously treated for nephrotic syndrome versus 30 healthy children as a control group. In their study, the inclusion required being in remission free of steroids for at least 4 years in contrary to our study which included children with NS on current treatment with steroids or other immunosuppressive agents. Also, Rahul et al. [16] reported no significant difference in the mean CIMT between cases and controls.

Our study revealed significantly higher mean CIMT in the SDNS group than other NS groups. Unlike our results, Ahmed et al. [18] did not find any significant difference in the CIMT between different steroid response groups. Also Rahul et al. [16] and Youssef et al. [17] found no significant difference in CIMT between patients with IFRNS, FRNS, SDNS, and SRNS. Paripović et al. [20] also reported no significant difference in CIMT between patients with SDNS and SRNS. The discrepancy between our study and those studies could be attribuated to the fact that the SDNS group had higher disease duration and number of relapses than other groups and since CIMT, as discussed later, was found to be positively correlated to disease duration and number of relapses, our study revealed higher CIMT in the SDNS group.

We didn’t find any significant difference in CIMT between different renal histopathological results. Similarly, Ahmed et al. [18] reported no difference in CIMT between children with MCD and FSGS.

In the current study, the mean CIMT was significantly higher among patients receiving non-steroid immunosuppressive therapy at the time of evaluation than those receiving steroids alone (*p*-value = 0.003). However, unlike our results, Paripović et al. [20] didn’t find any significant difference in the mean CIMT between these 2 groups of patients.

There was also a statistically significant higher mean CIMT among cases who received combined or serially immunosuppressive agents of CYP, CNI & MMF (*p*-value = 0.04). It appears from our data that the mean CIMT becomes progressively higher with combination of immunosuppressive therapy. However, more number of cases have to be included in further studies to confirm this assumption.

We didn’t find significant difference in CIMT between patients with nephrotic syndrome receiving CNI (*n* = 17) and those not receiving CNI (*n* = 23) (0.492 ± 0.05 versus 0.466 ± 0.04 respectively; *P*-value = 0.07). In consonance with our results, other studies [16, 20] didn’t find significant difference in CIMT between these 2 groups.

In our study, there was a significant positive correlation between CIMT and disease duration. Similar to our results, Hooman et al. [21] found that CIMT was correlated to disease duration longer than 2 years. Also other studies [10, 18, 20, 21, 26] reported similar results.

The number of relapses in our study showed a statistically significant positive correlation with CIMT. Other studies [10, 26] had also reported similar results but no correlation was reported by Paripović et al. [20].

In our study, CIMT was also positively correlated to advancing patients’ age *(r = 0.45, p = 0.004)* which is in consonance with Mehta et al. [26] study. A negative, but statistically insignificant correlation was noted between CIMT and serum albumin. Mehta et al. [26] found the similar finding. There was a significant positive correlation between CIMT and BMI. Litwin et al. [28] and Paripović et al. [20] reported a similar finding. However, unlike our study, There was no correlation between CIMT and BMI in Mehta et al. [26] study.

Development of early atherosclerosis in children with nephrotic syndrome could be attributed to long periods of dyslipidemia even during remission of nephrotic syndrome [10]. When CIMT was compared with total serum cholesterol and TG levels, we found no correlation between them. Similar findings were reported by other studies [17, 18, 20, 21, 29, 30]. Also, Mehta et al. [26] found no correlation of CIMT with LDL, HDL, triglyceride, and VLDL, however a statistically insignificant negative correlation with total serum cholesterol was found. However, other studies [10, 22, 31] found that total serum cholesterol, LDL cholesterol, and serum triglyceride level had positive and significant correlation with CIMT.

Systemic hypertension is a risk factor for the development of renal injury and cardiovascular disease [32]. In the present study there was a significant positive correlation between CIMT and diastolic blood pressure. However, CIMT was not correlated to systolic blood pressure. Chaubey et al. [22] showed a significant positive correlation between systolic blood pressure, diastolic blood pressure and CIMT. Ahmed et al. [18] had not found any correlation of CIMT to blood pressure, and this could be because only 12.2% of their NS children were hypertensive. Also, Hooman et al. [21] had shown weak positive correlation between blood pressure and CIMT.

Paripović et al. [20] reported a link between CIMT and ambulatory blood pressure parameters (both daytime and night-time systolic blood pressure), while there was no significant association with office blood pressure. This is consistent with a study by Flynn et al. [33] showing that ambulatory blood pressure monitoring provides superior assessment of blood pressure in comparison with office measurements.

The main limitation of this study is the lack of long-term follow up to see the changes in CIMT over a period of time and lack of studying the effect of cumulative dose of steroids on CIMT. We did not investigate carotid function including distensibility, stiffness and elasticity.

## Conclusion

In conclusion, CIMT was significantly increased in children with NS. There was significant positive correlation between CIMT and duration of the disease, number of relapses, body mass index, diastolic hypertension and advancing age. However, it’s not correlated to total serum cholesterol or triglycerides level. CIMT was significantly higher among patients receiving non-steroid immunosuppressive therapy than those receiving steroids alone. As increased CIMT is a surrogate marker of atherosclerosis in children, the assessment of CIMT can be of benefit on the long term care of patients with NS.

## Data Availability

The datasets used and/or analyzed during the current study are available from the corresponding author on reasonable request.

## Abbreviations

ACR: Albumin/creatinine ratio in urine
BMI: Body mass index
CIMT: Carotid intima media thickness
CYP: Cyclophosphamide
CNI: Calcineurin inhibitors
FRNS: Frequent relapse nephrotic syndrome
FSGS: Focal segmental glomerulosclerosis
GFR: Glomerular filtration rate
GN: Glomerulonephritis
IFRNS: Infrequent relapse nephrotic syndrome
MCD: Minimal change disease
MMF: Mycophenolate mofetil
NS: Nephrotic Syndrome
SDNS: Steroid dependent nephrotic syndrome
SRNS: Steroid resistant nephrotic syndrome
SSNS: Steroid sensitive nephrotic syndrome
TG: Triglycerides.
